# Effects of various exercise therapies on 6-min walk distance in patients with stable chronic obstructive pulmonary disease: a systematic review and network meta-analysis

**DOI:** 10.3389/fmed.2025.1668578

**Published:** 2025-10-29

**Authors:** Haoran He, Zhikai Qin, Kuiliang Liu, Guilan Shen

**Affiliations:** ^1^Beijing Union University, Beijing, China; ^2^Capital University of Physical Education and Sports, Beijing, China; ^3^School of Physical Education and Sport Science, Fujian Normal University, Fuzhou, Fujian, China

**Keywords:** chronic obstructive pulmonary disease, 6-min walk distance, exercise therapy, network meta-analysis, water-based exercise, Chinese traditional mind-body training

## Abstract

**Purpose:**

This study aimed to compare the effects of various exercise interventions on the 6-min walk distance (6MWD) in patients with stable chronic obstructive pulmonary disease (COPD) using network meta-analysis and to explore their optimal dosage configurations.

**Methods:**

Randomized controlled trials (RCTs) on exercise interventions for patients with COPD published before January 1, 2025, were retrieved from CNKI, PubMed, PsycINFO, Web of Science, and the Cochrane Library. A total of 55 eligible RCTs were included. A meta-analysis and network meta-analysis (using R software) evaluated the effects of water-based exercise, traditional Chinese mind-body training, and general exercise on 6MWD. For the optimal intervention method, subgroup analysis of the conventional meta-analysis and nonlinear meta-regression were used to examine the dosage-efficacy relationship.

**Results:**

Network meta-analysis showed water-based exercise (WBE) most favorably improved 6MWD (SUCRA = 98.9, MD = 83.91, 95% CI: 55.98–111.83), significantly outperforming traditional Chinese mind-body training (CTMBT) and general exercise (GE). Nonlinear meta-regression linked efficacy to dosage, with optimal outcomes achieved with ≥3 sessions/week (5 sessions as ideal), ≥60 min/session (90 min as perfect), an 8-weeks duration, and a total of 270 min/week. Patients aged ≤ 61.67 years showed more pronounced improvements.

**Conclusion:**

Water-based exercise was the most effective intervention for improving the 6MWD of COPD patients. Optimal results required managing frequency (≥3 times/week, ideally 5 times/week), session duration (≥60 min, ideally 90 min), 8 weeks, and a total of 270 min/week. Older patients (≤61.67 years) benefited more. This study supported individualized, evidence-based exercise rehabilitation strategies for COPD.

**Systematic review registration:**

https://www.crd.york.ac.uk/PROSPERO/view/CRD42025636763, identifier CRD42025636763.

## Introduction

Chronic obstructive pulmonary disease (COPD) is a common, preventable, and treatable chronic airway disease primarily characterized by persistent respiratory symptoms and airflow limitation due to structural abnormalities of the airways. These airway changes are typically caused by long-term exposure to harmful gases or particulate matter, with smoking being the most significant risk factor (1). Smoking is widely recognized as the primary cause of COPD, as it induces chronic inflammation and damages lung tissue, resulting in conditions such as chronic bronchitis and emphysema, both of which are key components of COPD (2). Notably, COPD has become the third leading cause of death worldwide, posing a serious threat to human health (1). Recent epidemiological studies indicate that the prevalence of COPD among individuals over the age of 40 varies significantly by gender and region, with overall prevalence being higher in men than in women. The highest rates are observed in the Americas and Southeast Asia, while the Eastern Mediterranean region reports the lowest prevalence. However, data gaps in Africa and the Eastern Mediterranean may affect the accuracy of global estimates (3). Furthermore, COPD is a systemic disease that not only impairs respiratory function but also negatively impacts multiple bodily systems. It can lead to systemic inflammation, nutritional depletion, and musculoskeletal dysfunction, reducing exercise tolerance (4). Due to this multisystem impairment, patients often experience a significant decline in exercise endurance, rendering physical activities in daily life increasingly challenging and severely impacting their quality of life (5). In addition to the typical respiratory symptoms, patients with COPD may experience extrapulmonary dysfunctions, such as balance abnormalities and an increased risk of falls (6). This phenomenon arises from the interaction between sensory input and motor function. In patients with COPD, impairments in the sensory system significantly exacerbate balance dysfunction. Challenges in postural control disrupt balance, further limiting their ability to perform daily activities (7). Regarding gait characteristics, patients typically exhibit compensatory changes, including a decrease in step frequency, a shortening of stride length, and an increase in the duration of double support, which are characteristic features of the condition (8). Furthermore, evidence suggests that these patients experience significant asynchronous movement between the abdominal wall and thoracic cage during breathing, which may further exacerbate their symptoms of dyspnea (9).

Studies have demonstrated that exercise interventions combining endurance and strength training can significantly enhance exercise tolerance in patients with COPD. These interventions are associated with increased walking distances and improvements in the 6-min walk distance (6MWD) test, underscoring the clinical importance of scientifically designed exercise programs in enhancing patients’ physical function (5). Exercise training induces various physiological adaptive responses, including muscle hypertrophy and neural adaptations associated with strength training, which enhance muscle strength and endurance, as well as cardiovascular improvements resulting from endurance training. These positive physiological changes contribute to improved oxygen delivery efficiency to working muscles (10). This enhancement in efficiency extends exercise duration, potentially reducing fatigue and dyspnea, thereby improving overall physical fitness (11, 12). Furthermore, exercise training influences the cytokine system and supports the neuroimmune state, enhancing cerebral blood flow and positively affecting metabolic regulation and neurohumoral changes (13, 14). Through these multifaceted mechanisms, exercise training effectively improves extrapulmonary function and gait stability in patients with COPD (15). Consequently, the comprehensive improvements by exercise training significantly enhance the overall physical capacity of COPD patients and improve their quality of life. This also provides an essential theoretical foundation and practical guidance for developing rehabilitation programs for COPD patients in clinical settings (5, 15).

Two meta-analyses focusing on interventions for patients with COPD specifically examined the effects of endurance training (ET) and the traditional Chinese exercise Baduanjin. The findings revealed that patients participating in ET and Baduanjin interventions exhibited significantly improved 6-min walk distance (6MWD) compared to the control group. This suggests that both endurance training and the traditional Chinese exercise, Baduanjin, are effective interventions for improving exercise function in patients with COPD. However, it is essential to note that the methodological quality of the clinical trials included in these analyses was generally low, underscoring the necessity for more rigorous randomized controlled trials to substantiate this conclusion (16, 17). Conversely, other studies have drawn different conclusions. A network meta-analysis indicated that pulmonary rehabilitation (PR) training programs conducted in urban settings yielded the most substantial effects. Upon comparing supervised PR, unsupervised PR, and home-based PR, the results demonstrated that supervised PR produced the most favorable intervention outcomes (18).

Although existing studies have confirmed the positive effects of exercise therapy on patients with COPD, there remains no consensus regarding the differential impacts of various exercise interventions on functional improvement (19). Traditional meta-analyses are limited by pairwise comparisons between interventions, which complicates the simultaneous assessment of multiple treatment options. In contrast, network meta-analysis synthesizes both direct and indirect evidence (20). The 6MWD is a widely utilized measure for assessing functional exercise capacity in patients with pulmonary diseases and possesses substantial prognostic predictive value. This measure is simple, cost-effective, and highly correlated with more complex assessment methods (21, 22). This study builds upon previous research in two significant ways: first, it incorporates randomized controlled trial data on newer interventions, such as water-based exercise; second, through a multidimensional evaluation, it examines the differences in the effects of various exercise modalities on the improvement of 6MWD in COPD patients, thereby providing more comprehensive evidence for clinical practice (23–30).

## Materials and methods

### Study design

This study presents a systematic review of randomized controlled trials (RCTs) conducted according to the Preferred Reporting Items for Systematic Reviews and Meta-Analyses (PRISMA) guidelines (31). Before screening the search results, the review protocol was registered with the International Prospective Register of Systematic Reviews (PROSPERO) under registration number CRD42025636763, ensuring adherence to the PRISMA statement throughout the review process.

### Study inclusion criteria

Studies were included if they were randomized controlled trials (RCTs) that involved multiple exercise interventions for patients with stable COPD, with a minimum intervention frequency of once per week. Eligible studies were required to compare exercise interventions with either a blank control or another form of exercise intervention. Participants in the included studies had to be adults (aged ≥ 18 years), regardless of sex, ethnicity, or socioeconomic status, who had been diagnosed with COPD. To be eligible, studies had to evaluate the 6MWD as the primary outcome. All included studies must be written in Chinese or English and provide full-text access. Narrative reviews, preclinical studies, duplicate publications, editorials, commentaries, gray literature, and conference abstracts were excluded from the analysis. Systematic reviews and study protocols were not eligible for inclusion; however, relevant systematic reviews were used for reference and cited when appropriate.

### Search strategy

This network meta-analysis aimed to evaluate the effects of different exercise interventions on the 6MWD in patients with stable COPD. To ensure a comprehensive and relevant collection of studies, a systematic search was conducted across the CNKI, PubMed, PsycINFO, Web of Science, and Cochrane Library databases, concluding on January 1, 2025. The search terms integrated keywords and subject headings, such as (“Pulmonary Disease, Chronic Obstructive” OR COPD OR “Chronic Obstructive Pulmonary Disease” OR “Stable COPD” OR “Stable Chronic Obstructive Pulmonary Disease”) AND (“Exercise Therapy” OR Exercise OR “Physical Activity” OR Training OR Rehabilitation) AND (“Walking Test” OR “6-min walk test” OR 6MWT OR “6 Min Walk”) AND (“Randomized Controlled Trial” OR RCT OR “Randomized Trial”). Detailed search strategies are provided in the Supplementary material.

### Study selection process

The search results were imported into Zotero 6.0. After removing duplicates, two reviewers independently screened the titles and abstracts of the studies. Studies that did not meet the eligibility criteria were excluded from the analysis. The full texts of all relevant studies were retrieved and downloaded for further assessment of eligibility. Any disagreements between the two reviewers regarding the inclusion of specific studies were resolved through consultation with a third independent reviewer to minimize bias in the selection process. The two reviewers independently extracted data from each study, and discrepancies were determined in consultation with the third reviewer.

### Data extraction

Data were extracted from the selected studies across several domains, including author, year of publication, sample size, participants’ age and sex, study design, description of the intervention (which encompasses intervention type, frequency, duration, and key components), control group, outcome measures and time points, results, dropout rates, and the handling of missing data.

### Effect size measurement

The outcome of the included studies was defined as the mean difference in exercise therapy between the intervention group and the control group at the final assessment. Two researchers independently extracted and recorded the data, and any discrepancies were resolved through consensus or consulting a third reviewer. Manuscripts were included in the meta-analysis only if 6MWD was reported as a primary outcome.

### Data synthesis

This study constructed a network meta-analysis model for continuous variables within a Bayesian framework to integrate both direct and indirect evidence, thereby evaluating the relative efficacy of multiple exercise interventions (32). The procedures were as follows: First, the treatment network was established using the gemtc package, which facilitated the visualization of node relationships and the analysis of fundamental network characteristics (33), such as closed loops and study connection density. Second, a consistency model (which assumed consistency between direct and indirect effect estimates) and an inconsistency model (the Unrelated Mean Effects, UME model) were developed. Both models employed a normal likelihood distribution, an identity link function, and a random-effects model to quantify heterogeneity (34). I^2^ quantifies the proportion of total variation due to heterogeneity, while τ^2^ estimates the variance of actual effects across studies (35, 36). Posterior sampling was conducted using the Markov Chain Monte Carlo (MCMC) algorithm, incorporating 3–4 independent chains, 20,000 burn-in samples, and 5,000–50,000 iterations to ensure convergence (37). Model convergence was evaluated using the Gelman-Rubin statistic (Rˇ < 1.05), trace plots, and posterior density plots. Model fit was compared using the Deviance Information Criterion (DIC), with a DIC difference greater than 3 indicating a significant difference in fit (38). Global consistency was evaluated by comparing the Deviance Information Criterion (DIC) values of the consistency model (consistency = “consistency”) and the inconsistency model (consistency = “ume”). A difference greater than 5 indicated notable heterogeneity. Additionally, node-splitting analysis (consistency = “node split”) was employed to identify sources of local inconsistency (39). The results presentation included: (1) Forest plots illustrating the mean difference (MD) and 95% credible intervals (CI) for each intervention compared to the control group; (2) Posterior ranking probabilities (posterior rank probs) and the surface under the cumulative ranking curve (SUCRA) to quantify the relative efficacy of interventions; and (3) A quadratic polynomial model mods = ∼ weekly time + weekly time sq to fit dose-response curves and identify optimal or suboptimal intervention dosages. In addition, for the best intervention identified, we conducted subgroup analyses of traditional meta - analysis. Since all studies used the same outcome scale, the effect size was calculated as the mean difference (MD) between groups:


$$MD\;=\;{\bar{X}}_{Intervention}-{\bar{X}}_{Control}\;,\;SD_{pooled}\;=$$

$$\sqrt{\frac{(n_{Intervention}\;-\;1)SD_{Intervention}^{2}\;+\;(n_{Control}\;-\;1)SD_{Control}^{2}}{n_{Intervention}\;+\;n_{Control}\;-\;2}}$$

(40). Sensitivity analyses encompassed convergence diagnostics (multi-core MCMC) and an assessment of evidence quality to ensure the robustness of the findings.

### Risk of bias (quality) assessment

The quality of each included study was assessed using the CINeMA tool, which evaluates the risk of bias in randomized trials (41). The assessment encompassed several domains: random sequence generation, allocation concealment, blinding of participants and personnel, blinding of outcome assessment, incomplete outcome data, selective reporting, and other sources of bias (42). Two independent authors conducted the quality assessments. Any disagreements were resolved through consultation with a third reviewer. The overall certainty of evidence for the optimal intervention was assessed using the GRADE approach, with the certainty of evidence evaluated according to the GRADE framework (43).

### Dose-response analysis of exercise interventions

This study employed a random-effects meta-regression model to quantify the relationship between exercise intervention dosage, specifically frequency, duration per session, total duration, and improvements in the 6-min walk distance (6MWD). Independent variables extracted from the intervention group of the included studies included exercise frequency (sessions per week), duration per session (minutes), total intervention period (weeks), and patient age (years), with the standardized mean difference (MD) serving as the dependent variable. A generalized linear model was constructed using the R programming language to examine both linear and nonlinear relationships of the dose-effect, including a quadratic term test. For significant nonlinear associations, such as total exercise duration per week, a restricted cubic spline curve was fitted to calculate the optimal dose range and 95% confidence interval.

## Results

### Study selection

**FIGURE 1:**
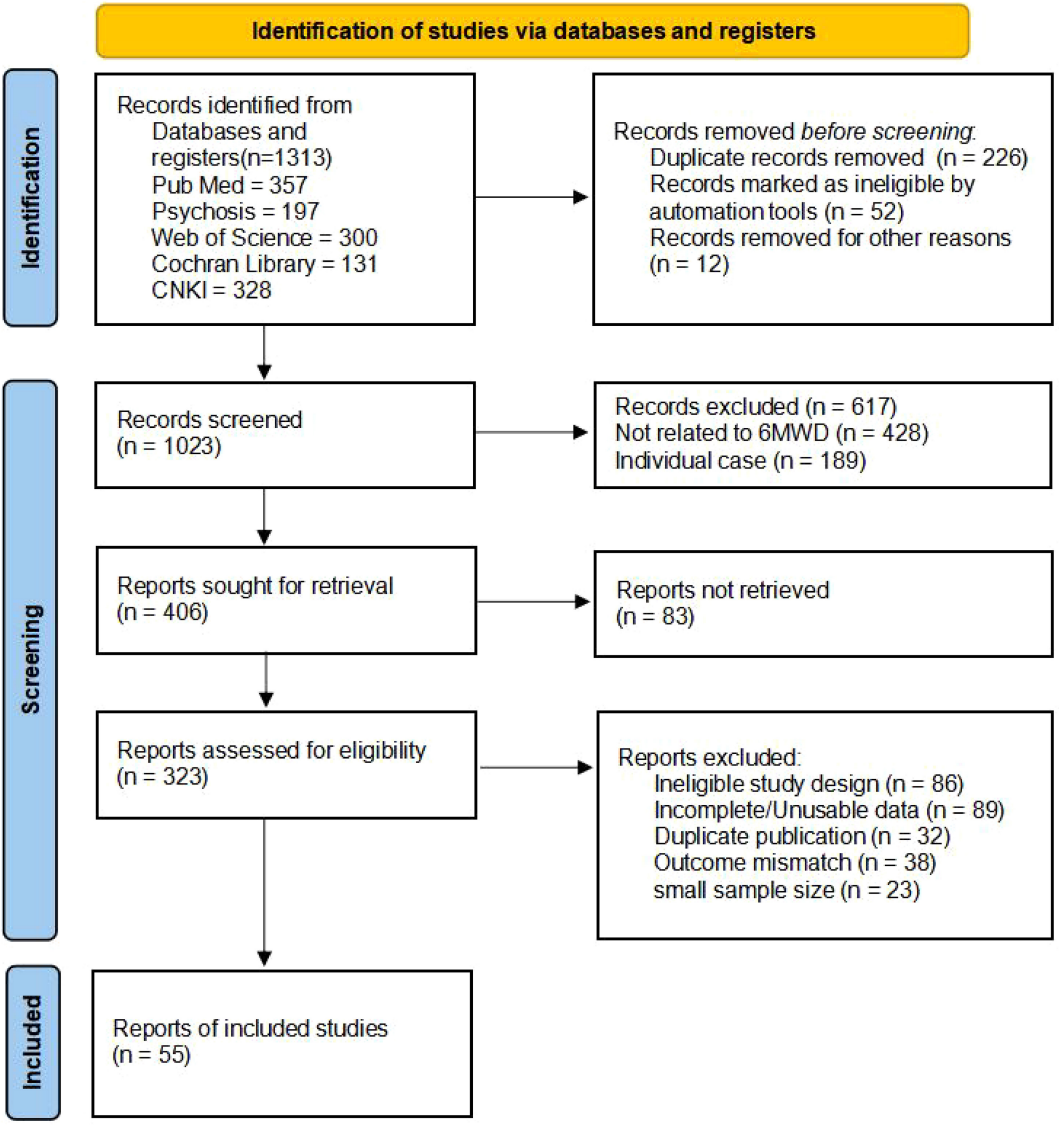
Flowchart of the study.

One thousand two hundred fifty articles were initially screened from four databases: CNKI, PubMed, PsycINFO, Web of Science and the Cochrane Library. Among these, 226 articles were identified as duplicates. After reviewing the titles and abstracts of the remaining 1,023 articles, 617 were excluded for not meeting the inclusion criteria. Consequently, 323 articles passed the initial screening and were subjected to a full-text review. After this further evaluation, 268 articles were excluded, resulting in a final inclusion of 55 studies for the network meta-analysis (see Figure 1).

### Risk of bias of included studies

**FIGURE 2:**
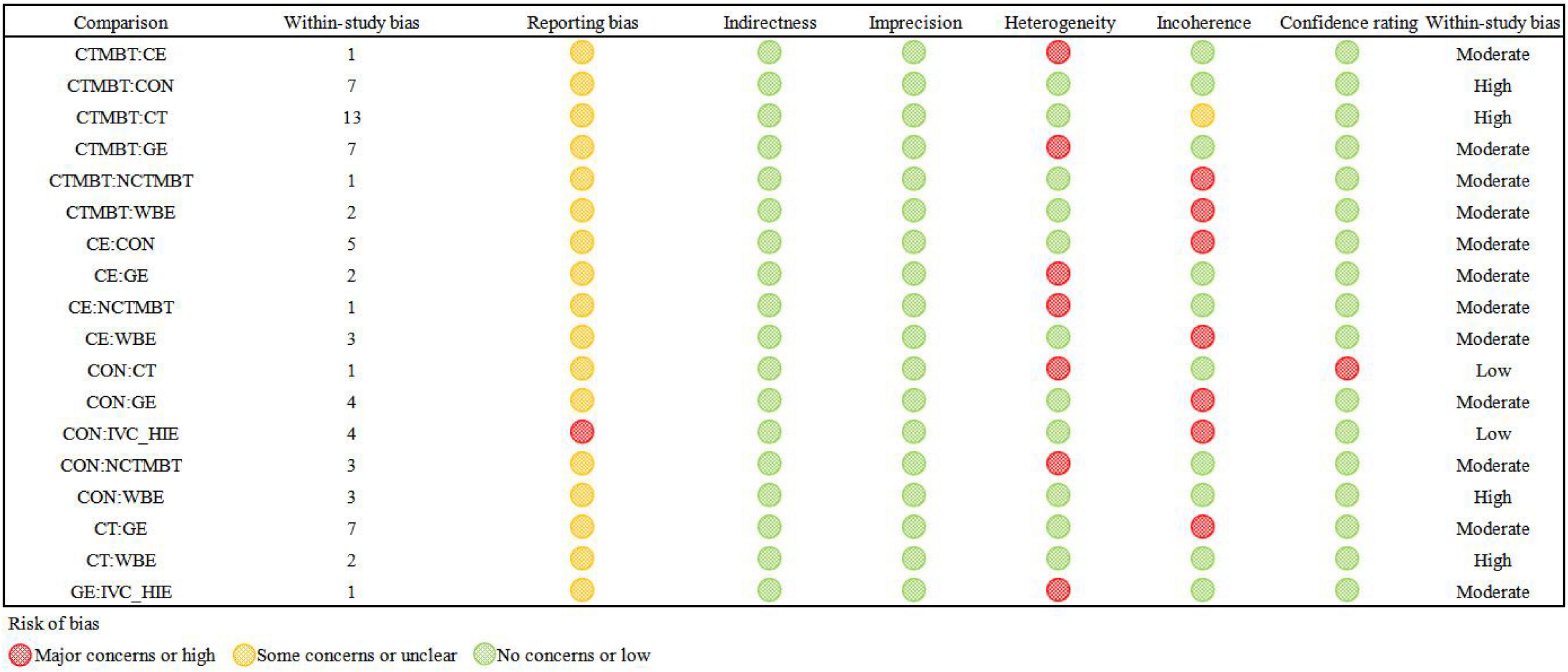
Risk of bias graph. CON, control; CT, conventional treatment; CTMBT, Chinese traditional mind-body training; NCTMBT, Non-Chinese Traditional Mind-Body Training; IVC-HIE, Interval Versus Continuous High Intensity Exercise; WBE, Water Based Exercise; CE, Combined Exercise; GE, General Exercise.

According to the Cinema methodology, evidence quality is categorized into four levels: (1) High quality, indicating that the actual effect size closely approximates the estimate derived from the network meta-analysis (NMA); (2) Moderate quality, suggesting that the actual effect size may be near the NMA estimate, yet significant differences may still exist; (3) Low quality, implying that the actual effect size could differ significantly from the NMA estimate; and (4) Very low quality, denoting that the actual effect size is likely to differ substantially from the NMA estimate. The certainty of evidence is evaluated using the online CINeMA software based on several criteria, including within-study bias, publication bias, indirectness, imprecision, heterogeneity, and inconsistency (41, 43). If any issues are identified during this assessment, the evidence level is downgraded accordingly (see Figure 2).

### Study characteristics

The main characteristics of these studies are detailed in Table 1. The controlled trials examined patients with stable COPD who were 18 years of age or older. Most participants were between 40 and 80, with several studies focusing on elderly patients. The interventions primarily encompassed Chinese Traditional Mind-Body Training, Non-Chinese Traditional Mind-Body Training, Interval Versus Continuous High-Intensity Exercise, Water-Based Exercise, Combined Exercise, and General Exercise. This study incorporated 55 randomized controlled trials (RCTs) that compared various exercise interventions. The sample sizes across these studies varied significantly, with the number of participants per group ranging from 10 to 70. Most studies included between 20 and 50 participants per group, and the duration of the interventions varied from 1 to 28 weeks. All included studies reported cost-related information, such as intervention delivery expenses, equipment use, or healthcare utilization.

**TABLE 1:** Characteristics of the randomized controlled trials included in patients with COPD.

References	Country	Sample size (T/C)	Age range	Intervention design (T/C)	Exercise delivery format	Exercise prescription	Evaluation tools/content	Significant improvement
Niu et al. (44)	China	20/19	≥45	CTMBT/CT	Group + individual	50 min/times, 7/week, 24 weeks	6MWD	Yes
Chan et al. (45)	China	70/67	55–58	CTMBT/CON	Group + individual	60 min/times, 2/week, 12 weeks	6MWD	Yes
Chan et al. (45)	China	70/69	55–58	CTMBT/GE	Group + individual	60 min/times, 2/week, 12 weeks	6MWD	Yes
Li et al. (46)	China	17/19	40–80	CTMBT/CT	Group + individual	60 min/times, 7/week, 24 weeks	6MWD	Yes
Xiao et al. (47)	China	59/60	65–85	CE/GE	Group + individual	45 min/times, 4/week, 24 weeks	6MWD	Yes
Wu et al. (48)	China	16/17	40–80	CTMBT/CT	Group + individual	60 min/times, 6/week, 24 weeks	6MWD	Yes
Wu et al. (48)	China	16/17	40–80	CTMBT/CE	Group + individual	60 min/times, 6/week, 24 weeks	6MWD	Yes
Fang et al. (49)	China	61/60	71.75 ± 9.38	CTMBT/NCTMBT	Group + individual	45 min/times, 7/week, 24 weeks	6MWD	Yes
Pan et al. (50)	China	23/23	45–75	CTMBT/CT	Group	45 min/times, 3/week, 8 weeks	6MWD	Yes
Chen et al. (51)	China	31/29	70.82 ± 9.50	CTMBT/CT	Not reported	30 min/times, 7/week, 12weeks	6MWD	Yes
Deng et al. (52)	China	27/27	64.4 ± 8.76	CTMBT/CON	Group + individual	45 min/times, 7/week, 24 weeks	6MWD	Yes
Zhang et al. (53)	China	30/30	65.14 ± 6.49	CTMBT/CT	Group + individual	45 min/times, 7/week, 24 weeks	6MWD	Yes
Liu et al. (54)	China	50/50	70.98 ± 10.79	CTMBT/CON	Group	45 min/times, 7/week, 12 weeks	6MWD	Yes
Du et al. (55)	China	36/38	65.24 ± 8.37	CTMBT/GE	Group + individual	60 min/times, 7/week, 12 weeks	6MWD	No
Du et al. (55)	China	36/38	65.24 ± 8.37	CTMBT/CON	Group + individual	60 min/times, 7/week, 12 weeks	6MWD	Yes
Gao et al. (56)	China	36/35	66.67 ± 8.65	CTMBT/GE	Individual	45 min/times, 2/week, 12 weeks	6MWD	Yes
Zhao et al. (57)	China	30/30	57.79 ± 6.22	CTMBT/CT	Group + individual	45 min/times, 7/week, 12 weeks	6MWD	Yes
Zhu et al. (58)	China	26/27	54.68 ± 10.19	CTMBT/GE	Group	45 min/times, 7/week, 12 weeks	6MWD	Yes
Zhu et al. (58)	China	26/21	54.68 ± 10.19	CTMBT/CT	Group	45 min/times, 7/week, 12 weeks	6MWD	Yes
Zhu et al. (59)	China	63/60	60.4 ± 11.0	CTMBT/CT	Individual	45 min/times, 2/week, 24 weeks	6MWD	Yes
Li et al. (60)	China	44/44	80.99 ± 4.22	CTMBT/CT	Individual	45 min/times, 7/week, 12 weeks	6MWD	Yes
Liu et al. (61)	China	51/32	61.64 ± 7.93	CTMBT/GE	Group + individual	60 min/times, 3/week, 24 weeks	6MWD	Yes
Liu et al. (61)	China	51/35	62.00 ± 7.09	CTMBT/CT	Group + individual	60 min/times, 3/week, 24 weeks	6MWD	Yes
Liu et al. (62)	China	25/26	45–75	CE/CON	Group + individual	45 min/times, 3/week, 8 weeks	6MWD	Yes
Liu et al. (62)	China	26/26	45–75	CTMBT/CON	Group + individual	45 min/times, 3/week, 8 weeks	6MWD	No
Ranjita et al. (63)	India	36/36	36–60	NCTMBT/CON	Group	90 min/times, 6/week, 12 weeks	6MWD	Yes
Puhan (64)	Switzerland	48/50	68.95 ± 9.2	IVC_HIE/GE	Group + individual	45 min/times, 4∼5/week, 3 weeks	6MWD	Yes
Hasanpour Dehkordi et al. (65)	Iranian	40/40	53.15 ± 8.1	NCTMBT/CON	Group + individual	45 min/times, /week, 12 weeks	6MWD	Yes
Dong et al. (66)	China	10/10	40–75	CTMBT/GE	Group	45 min/times, 2/week, 12 weeks	6MWD	Yes
Ng et al. (67)	China	40/40	72.44 ± 1.20	CTMBT/CT	Group + individual	12–15 min/times, 7/week, 24 weeks	6MWD	No
Kaminsky et al. (68)	USA	21/22	68 ± 8.1	NCTMBT/CON	Group + individual	60 min/times, 2/week, 6 weeks	6MWD	No
Cameron-Tucker et al. (69)	Australia	42/40	65.8 ± 9.35	GE/CT	Group + individual	60 min/times, 1/week, 6 weeks	6MWD	Yes
Yu et al. (70)	China	35/37	68.7 ± 4.54	GE/CT	Group	45 min/times, 12/week, 2 weeks	6MWD	Yes
Jiang et al. (71)	China	18/19	40–80	CTMBT/CT	Group + individual	60 min/times, 14/week, 12 weeks	6MWD	Yes
de Souto Araujo et al. (30)	Brazil	13/11	63.16 ± 11.2	GE/CON	Group	90 min/times, 3/week, 8 weeks	6MWD	No
de Souto Araujo et al. (30)	Brazil	8/11	63.16 ± 11.2	WBE/CON	Group	90 min/times, 3/week, 8 weeks	6MWD	Yes
Pleguezuelos et al. (72)	Spain	37/54	71.03 ± 2.52	CT/CON	Individual	70 min/times, 3/week, 18 weeks	6MWD	Yes
Pleguezuelos et al. (72)	Spain	34/54	71.03 ± 2.52	GE/CON	Individual	70 min/times, 3/week, 18 weeks	6MWD	Yes
Kawagoshi et al. (73)	Japan	12/15	74 ± 8	GE/CT	Individual	720 min/times, 7/week, 18 weeks	6MWD	Yes
Breyer et al. (74)	Australia	30/30	62 ± 9	GE/CON	Group	60 min/times, 3/week, 12 weeks	6MWD	Yes
Cruz et al. (75)	Portugal	16/16	66.5 ± 8.4	GE/CT	Group + individual	60 min/times, 3/week, 12 weeks	6MWD	Yes
Hospes et al. (76)	Netherlands	18/17	45–75	GE/CT	Individual	20 min/times, 7/week, 12 weeks	6MWD	No
Mendoza et al. (77)	Chile	52/50	68.7 ± 8.5	GE/CON	Individual	45 min/times, 7/week, 12 weeks	6MWD	Yes
Probst et al. (78)	Brazil	20/20	68.24 ± 10.98	NCTMBT/CE	Group	60 min/times, 3/week, 12 weeks	6MWD	Yes
Liu et al. (29)	China	15/16	54–76	CTMBT/CON	Group	60 min/times, 2/week, 12 weeks	6MWD	Yes
Liu et al. (29)	China	14/16	54–76	WBE/CON	Group	60 min/times, 2/week, 12 weeks	6MWD	Yes
Liu et al. (29)	China	14/16	54–76	CTMBT/WBE	Group	60 min/times, 2/week, 12 weeks	6MWD	Yes
Redwood and Almeida (5)	Portugal	25/25	67.2 ± 8.5	CE/CON	Group	30 min/times, 3/week, 12 weeks	6MWD	Yes
Elmorsy et al. (79)	Egypt	19/18	63.59 ± 9.47	CE/CON	Group	60 min/times, 3/week, 8 weeks	6MWD	Yes
Zare et al. (80)	Iran	16/16	62.53 ± 9.21	CE/CON	Group	8 min/times, 10/week, 1 week	6MWD	Yes
Fastenau et al. (81)	Netherlands	46/44	62.5 ± 9.9	GE/CT	Group + individual	60–90 min/times, 5/week, 16 weeks	6MWD	Yes
Wootton et al. (82)	Australia	62/39	68.61 ± 8.41	GE/CT	Group	30∼45 min/times, 2∼3/week, 8∼10 weeks	6MWD	Yes
Alcazar et al. (83)	Spain	14/15	78.79 ± 7.24	IVC_HIE/CON	Group	2/week, 12 weeks	6MWD	No
Boeselt et al. (84)	Germany	20/17	65.73 ± 8.3	IVC_HIE/CON	Group	3/week, 24 weeks	6MWD	Yes
Hsieh et al. (85)	China	16/18	72.10 ± 7.12	IVC_HIE/CON	Group	2/week, 6 weeks	6MWD	Yes
Mador et al. (86)	USA	21/20	71.81 ± 7.42	IVC_HIE/CON	Group	3/week, 8 weeks	6MWD	Yes
Dourado et al. (87)	Brazil	11/13	63.89 ± 9.71	CE/GE	Group	60 min/times, 3/week, 12 weeks	6MWD	Yes
Felcar et al. (28)	Brazil	20/16	68.56 ± 8.58	WBE/CE	Group	60min/times, 2/week, 12weeks	6MWD	Yes
Rinaldo et al. (88)	Italy	14/14	66.15 ± 4.35	CE/CON	Group + individual	60 min/times, 1‘3/week, 28 weeks	6MWD	Yes
de Castro et al. (27)	Brazil	14/17	64.5 ± 8	WBE/CE	Group	60 min/times, 3/week, 12 weeks	6MWD	Yes
Gallo-Silva et al. (25)	Brazil	10/9	66.5 ± 9.5	WBE/CT	Individual	60 min/times, 3/week, 8 weeks	6MWD	Yes
Gallo-Silva et al. (26)	Brazil	11/11	65.9 ± 8.3	WBE/CON	Individual	60 min/times, 3/week, 8 weeks	6MWD	Yes
Hu et al. (24)	China	18/20	60–75	WBE/CT	Group	30 min/times, 3/week, 24 weeks	6MWD	Yes
Hu et al. (24)	China	18/18	60–75	WBE/CTMBT	Group	30 min/times, 3/week, 24 weeks	6MWD	Yes
Wang et al. (23)	China	25/25	61.67 ± 2.72	WBE/CE	Individual	30 min/times, 5/week, 8 weeks	6MWD	Yes

T, intervention group; C, control group; RCT, randomized controlled trial; COPD, chronic obstructive pulmonary disease; CON, control; CT, conventional treatment; CTMBT, Chinese traditional mind-body training; NCTMBT, Non-Chinese Traditional Mind-Body Training; IVC-HIE, Interval Versus Continuous High Intensity Exercise; WBE, Water Based Exercise; CE, Combined Exercise; GE, General Exercise; 6MWD, 6-min walk distance.

### Meta-analysis

**FIGURE 3:**
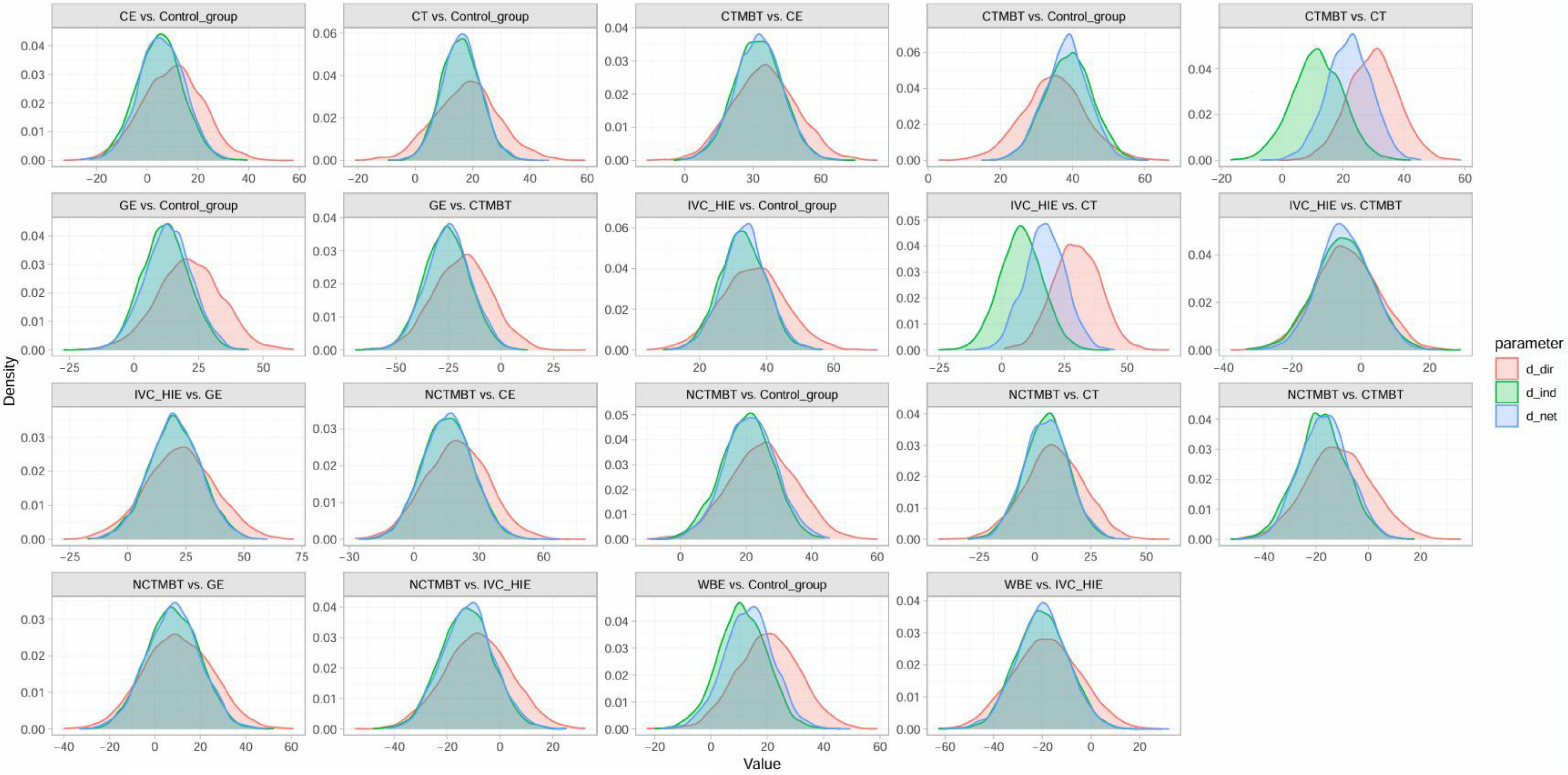
Node splitting method. CON, control; CT, conventional treatment; CTMBT, Chinese traditional mind-body training; NCTMBT, Non-Chinese Traditional Mind-Body Training; IVC-HIE, Interval Versus Continuous High Intensity Exercise; WBE, Water-Based Exercise; CE, Combined Exercise; GE, General Exercise.

The six interventions examined in this study included Chinese Traditional Mind-Body Training, Non-Chinese Traditional Mind-Body Training, Interval Versus Continuous High-Intensity Exercise, Water-Based Exercise, Combined Exercise, and General Exercise. A global inconsistency test was conducted, yielding a result (*P* = 0.80) that was greater than 0.05. A local inconsistency test using the node-splitting method also demonstrated that all *P*-values exceeded 0.05 (refer to Figure 3). The model fitting results indicated that the deviance information criterion (DIC) for the consistency model was 134.20, while the DIC for the inconsistency model was 133.35, resulting in a difference of 0.85 (less than 5), which suggests a good model fit. A random-effects model was used to pool the results.

**FIGURE 4:**
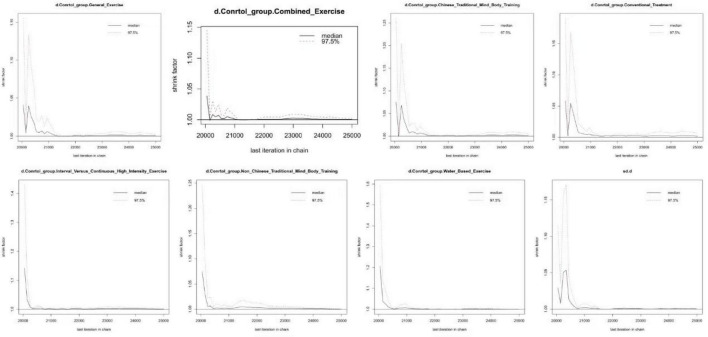
Convergence diagnostic plots.

**FIGURE 5:**
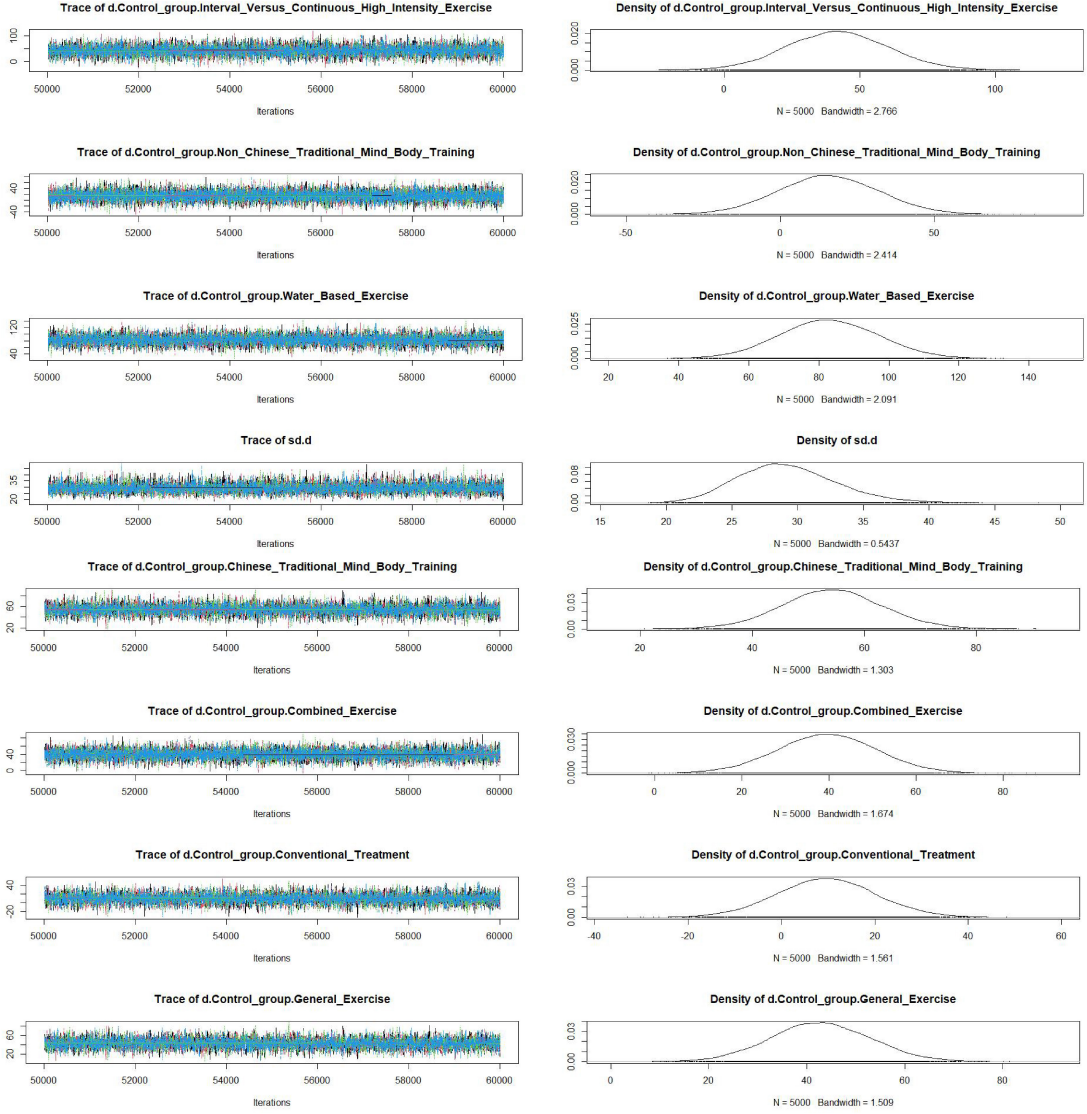
Trace plots and density plots.

The convergence diagnostic plot illustrated the successful convergence of the Markov Chain Monte Carlo (MCMC) simulation. The trajectories of each chain closely overlapped, and the posterior density distribution appeared smooth and symmetric. This indicated stable parameter estimates without noticeable divergence or oscillation, confirming the model’s validity and reliability (see Figure 4). The model fit was satisfactory, with a deviance information criterion (DIC) of 250.6 (Dbar = 134.4, pD = 116.2), and a data-to-model fit ratio of 1.003, indicating adequate model performance. In the trace and density plots, the fluctuations of each chain were consistent and tended to stabilize. Additionally, the density plot exhibited a unimodal distribution with no significant skewness, further corroborating the convergence and consistency of the model parameters (see Figure 5). A random-effects model was used to pool the results. The network results Showed That Between-Study heterogeneity was very low (I^2^ = 1%; τ≈ 29.2). After performing a consistency check on the involved studies and conducting a sensitivity analysis, all other studies fell within a reasonable range, as detailed in the Supplementary file.

**FIGURE 6:**
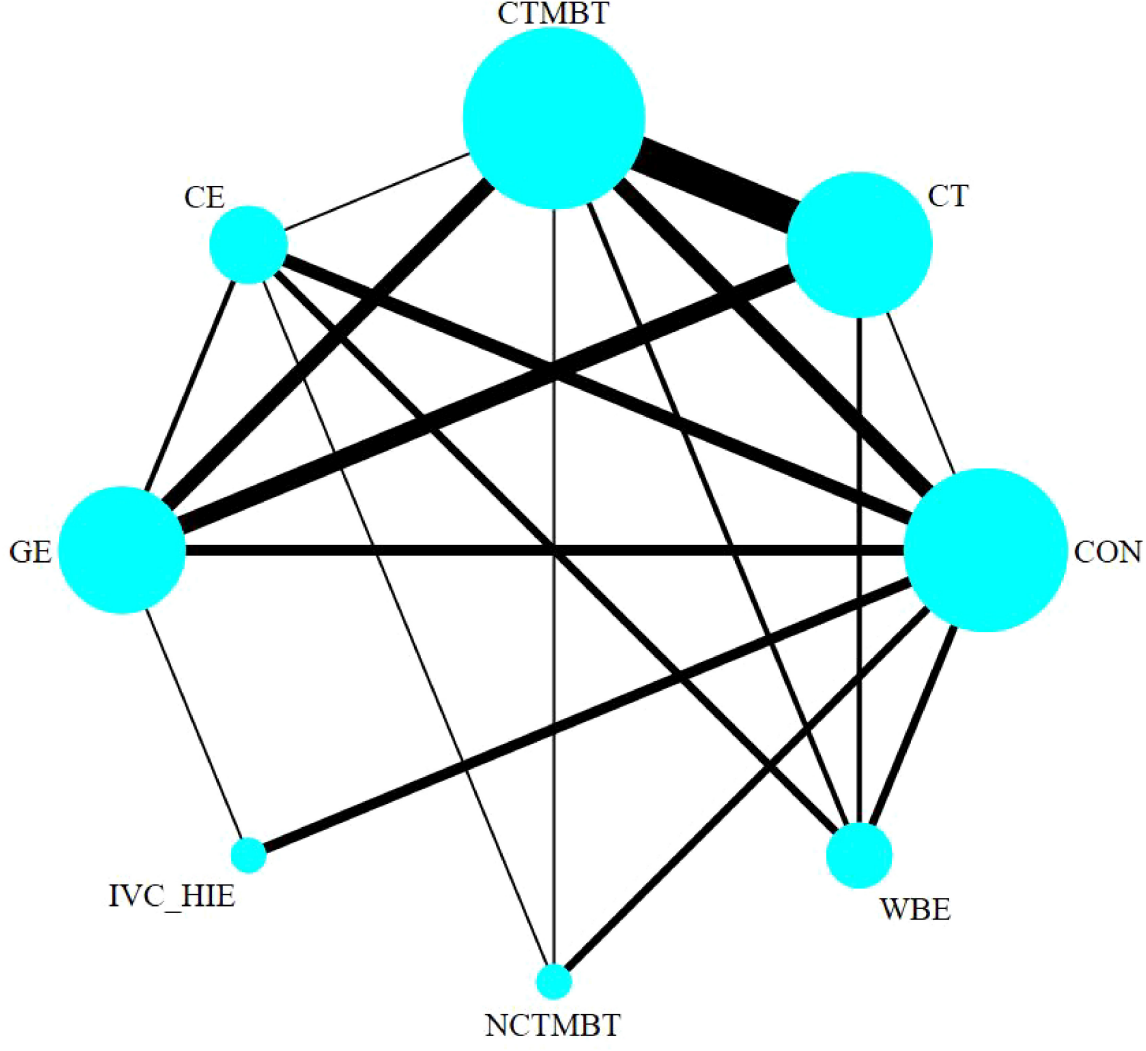
Evidence network for six types of exercise therapy to intervene in the 6MWD of patients with COPD. CON, control; CT, conventional treatment; CTMBT, Chinese traditional mind-body training; NCTMBT, Non-Chinese Traditional Mind-Body Training; IVC-HIE, Interval Versus Continuous High Intensity Exercise; WBE, Water Based Exercise; CE, Combined Exercise; GE, General Exercise.

Thus, it indicates that the direct and indirect effects of various exercise therapies compared to the control group on 6MWD in stable chronic obstructive pulmonary disease patients were consistent, and statistical analysis should be conducted under the consistency model (see Figure 6).

### Publication bias or small-study effects test

**FIGURE 7:**
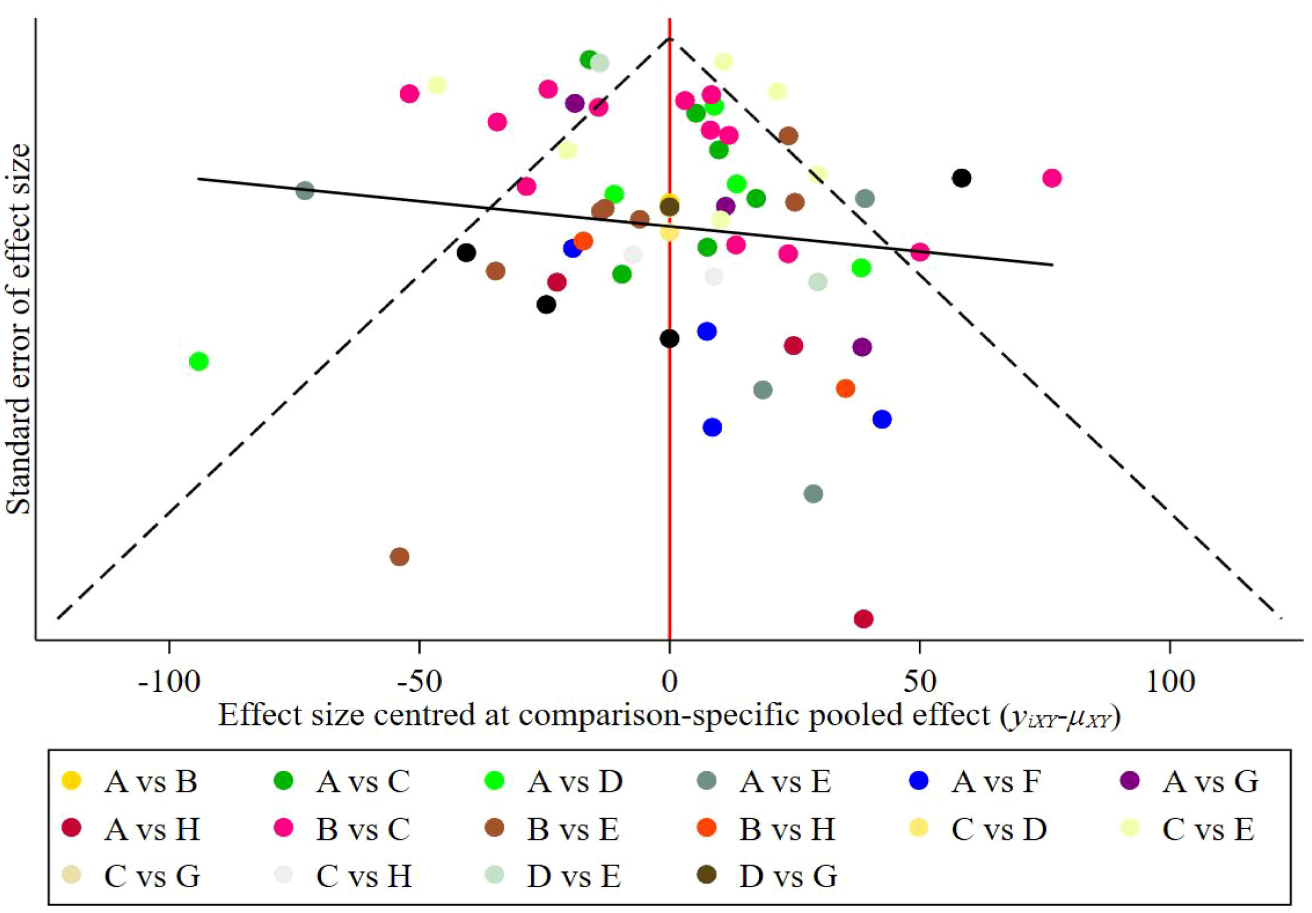
Publication bias funnel plot. A: control; B: conventional treatment; C: Chinese traditional mind-body training; D: Combined Exercise; E: General Exercise; F: Interval Versus Continuous High Intensity Exercise; G: Non-Chinese Traditional Mind-Body Training; H: Water Based Exercise.

Publication bias detection for the studies on the six exercise therapies showed that the included studies were generally symmetrical, suggesting that the possibility of publication bias or minor sample effects in the current research is relatively low (see Figure 7).

### Network meta-analysis results interpretation

**FIGURE 8:**
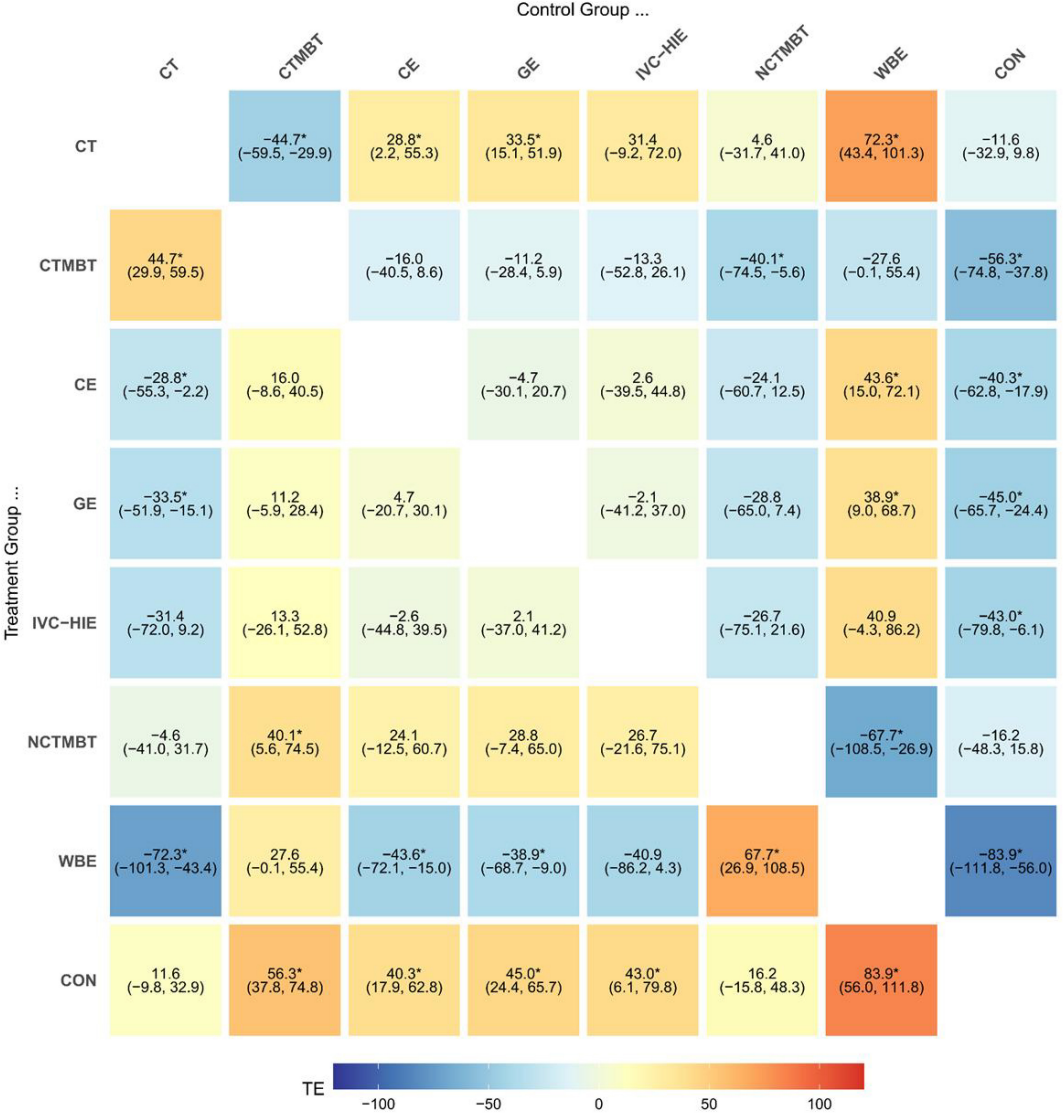
Mesh meta-analysis of 6MWD. An asterisk (*) indicates that the difference between the two interventions is statistically significant (*P* < 0.05). The color gradient represents the treatment effect (TE), where blue indicates negative effects, red indicates positive effects, and lighter colors indicate smaller effects. CON, control; CT, conventional treatment; CTMBT, Chinese traditional mind-body training; NCTMBT, Non-Chinese Traditional Mind-Body Training; IVC-HIE, Interval Versus Continuous High Intensity Exercise; WBE, Water-Based Exercise; CE, Combined Exercise; GE, General Exercise.

**FIGURE 9:**
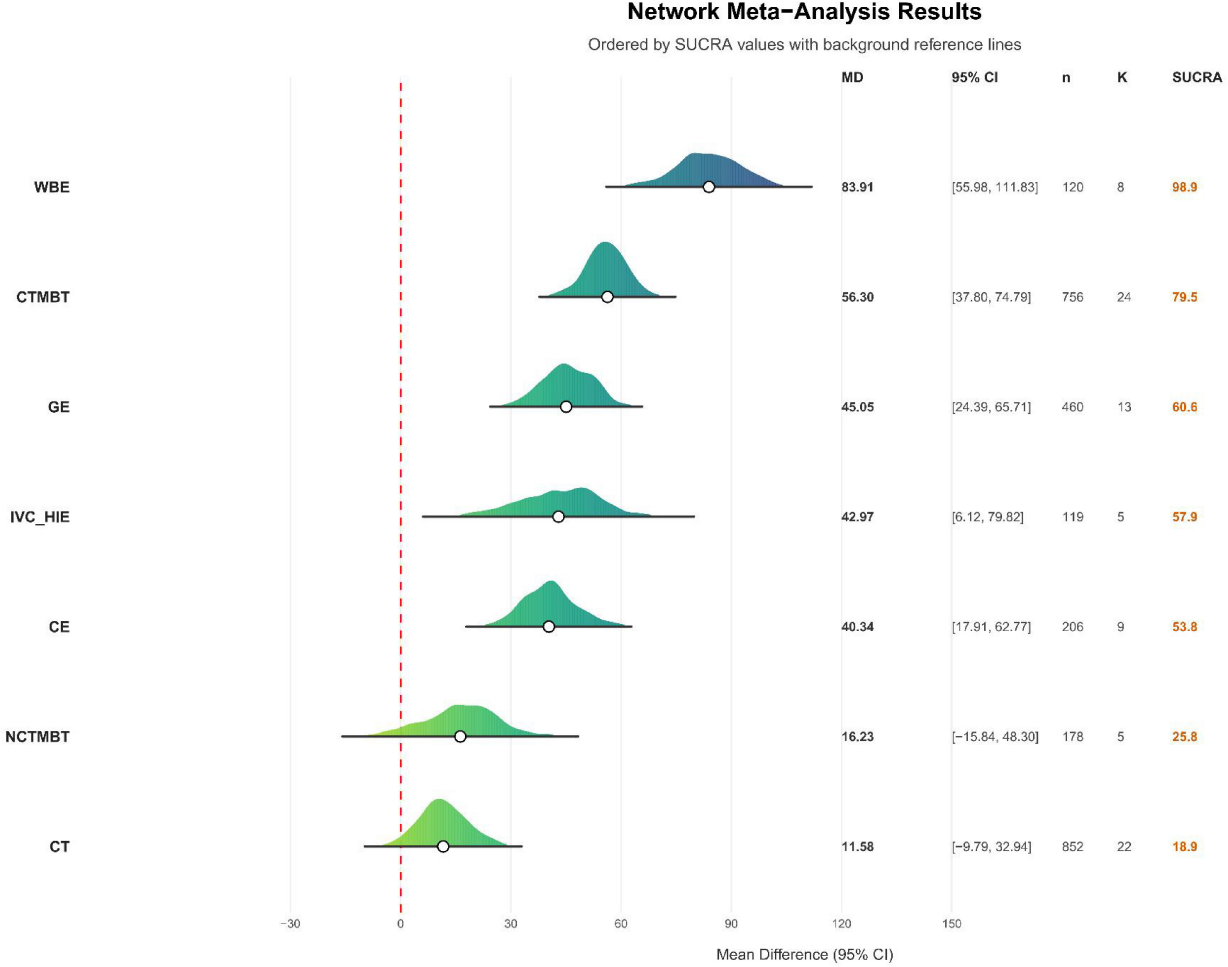
Direct comparison of cumulative probabilities between different intervention methods. CT, conventional treatment; CTMBT, Chinese traditional mind-body training; NCTMBT, Non-Chinese Traditional Mind-Body Training; IVC-HIE, Interval Versus Continuous High Intensity Exercise; WBE, Water Based Exercise; CE, Combined Exercise; GE, General Exercise; SUCRA, Surface Under the Cumulative Ranking Curve represents the probability that each intervention is the best among all evaluated options.

The direct and indirect comparison results are presented below (see Figure 8). The outcome measure included the 6MWD, expressed in meters, which resulted in relatively large effect sizes. The network results indicated that the Surface Under the Cumulative Ranking Curve (SUCRA) for various exercise therapies, ranked from highest to lowest, are as follows: Water-Based Exercise (98.9%), Chinese Traditional Mind-Body Training (79.5%), General Exercise (60.6%), Interval Versus Continuous High-Intensity Exercise (57.9%), Combined Exercise (53.8%), Non-Chinese Traditional Mind-Body Training (25.9%), Conventional Treatment (18.9%), and Control (4.6%) (see Figure 9). SUCRA values range from 0% to 100%. The higher the SUCRA value, and the closer to 100%, the higher the likelihood that a therapy is in the top rank or one of the top ranks; the closer to 0 the SUCRA value, the more likely that a treatment is in the bottom rank, or one of the bottom ranks (89).

### Nonlinear meta-regression results

**FIGURE 10:**
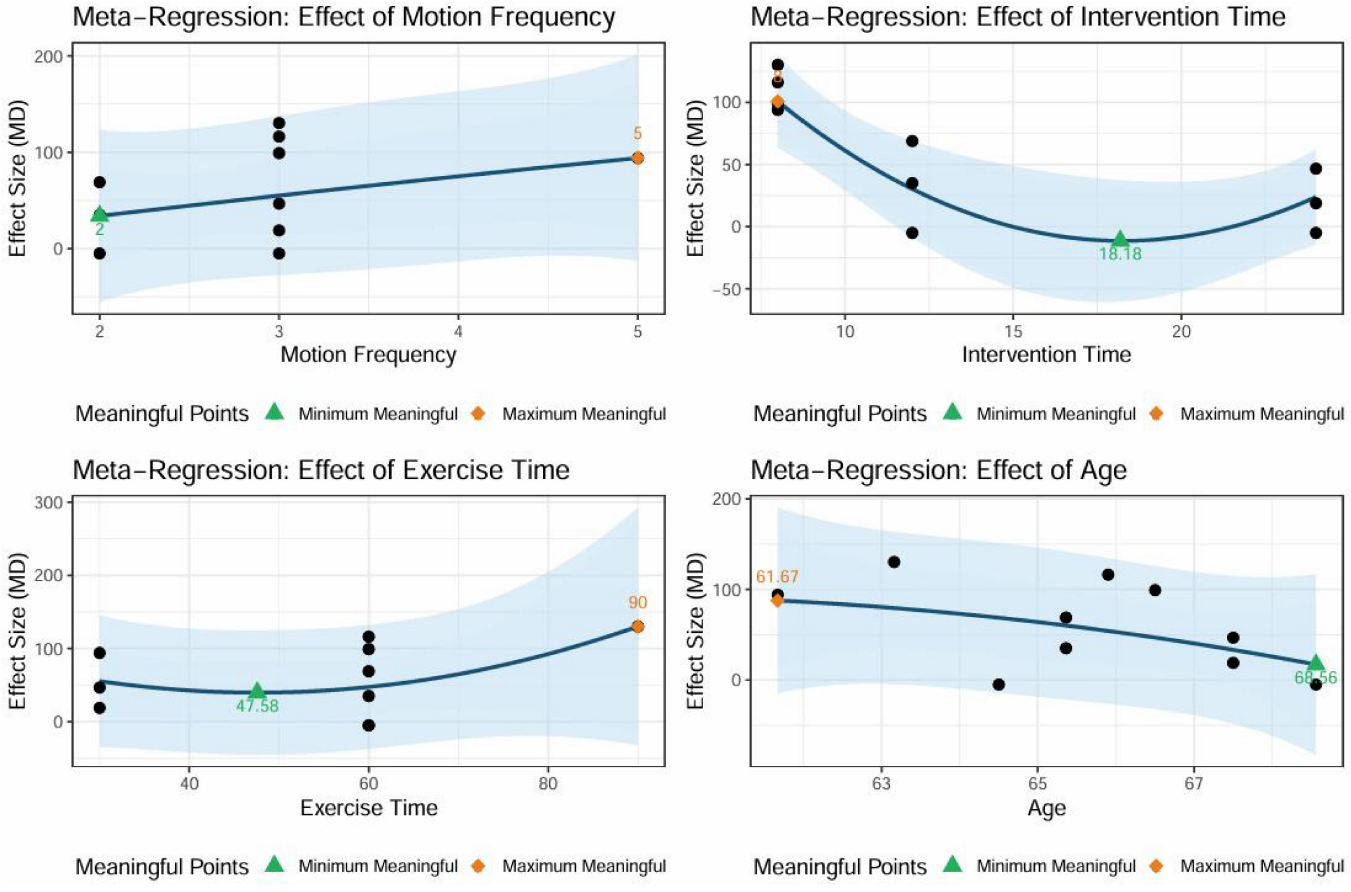
Meta-regression analysis of exercise intervention dose parameters and improvement in 6MWD. Units: motion frequency (sessions/week), intervention time (weeks), exercise time (minutes), age (years).

**FIGURE 11:**
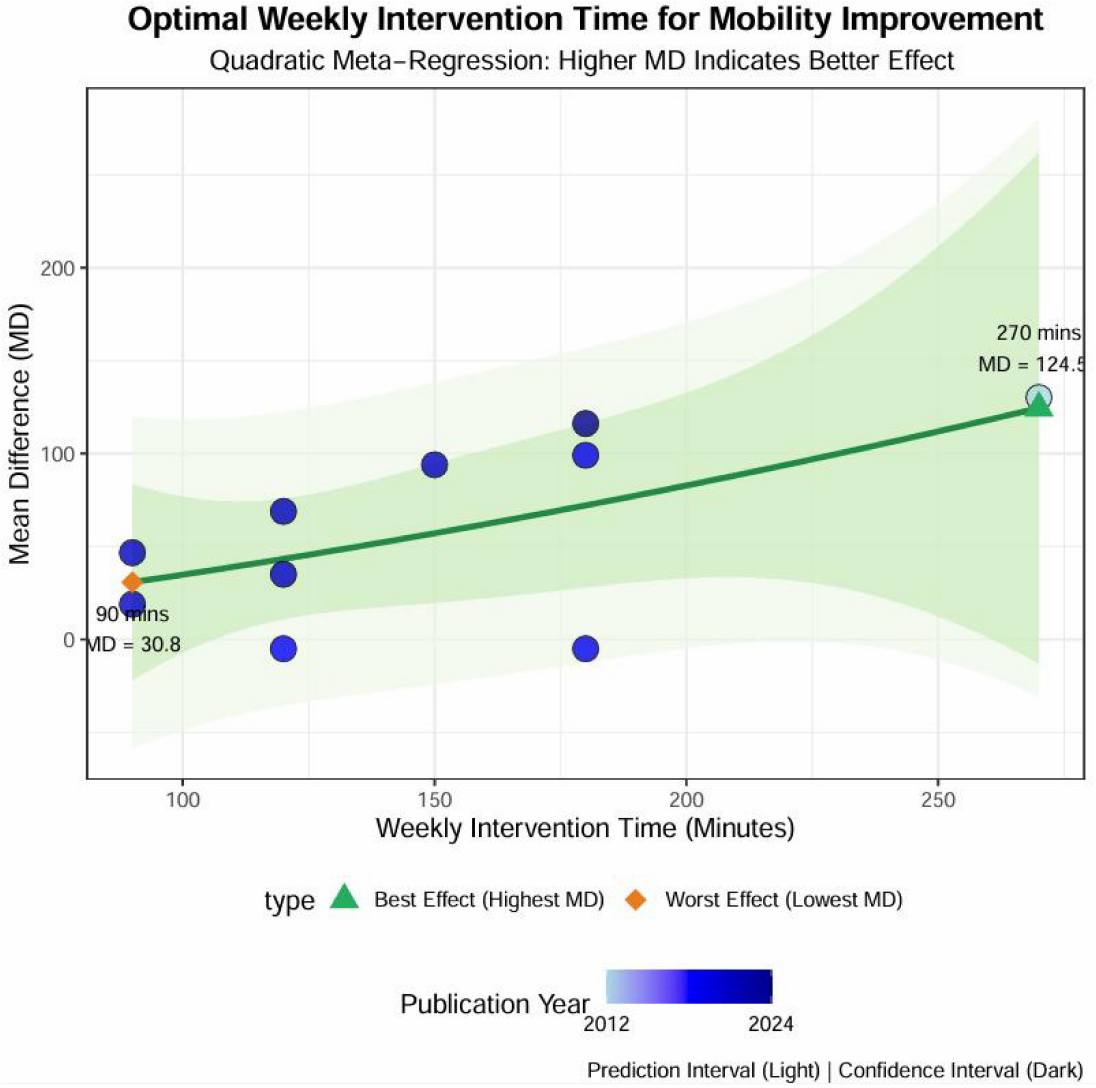
Nonlinear relationship between total weekly exercise duration and improvement in 6MWD.

The nonlinear regression results indicated a significant association between the exercise intervention dosage parameters and improvements in the 6MWD (refer to Figure 10). An exercise frequency of at least three sessions per week was sufficient to enhance the intervention’s effectiveness, with five sessions per week identified as the optimal frequency predicted by the model. Regarding session duration, engaging in exercise for a minimum of 60 min improved functional outcomes, while 90 min was identified as the ideal peak duration, as indicated by the graph. The analysis of intervention duration revealed that an 8-weeks intervention period yielded the most favorable results. Age analysis suggested that the intervention effect was more pronounced in older patients aged 61.67 years or younger. The results of the dosage meta-analysis demonstrated a clear dose-response relationship between the weekly intervention time and its effectiveness (see Figure 11). The optimal intervention dosage was determined to be 270 min per week. The trend prediction results based on the regression model are illustrated in the graph; however, these do not represent definitive intervention standards.

### Traditional meta-analysis results

In addition to the network meta-analysis and nonlinear dose–response modeling, we performed a traditional pairwise meta-analysis focusing on Water-Based Exercise (WBE), which was identified as the most effective intervention in improving 6MWD. The overall pooled analysis indicated that WBE produced clinically meaningful improvements in walking capacity compared with control interventions (see Supplementary Documentary). The analysis showed substantial heterogeneity (I^2^ = 66.7%, τ^2^ = 1222.6934, *p* = 0.0014).

To further explore potential effect modifiers, subgroup analyses were conducted based on age, race, motion frequency, intervention time, and exercise time per session. (see Supplementary Documentary).

Subgroup analyses were performed to explore potential effect modifiers.

The following presents the effect size and heterogeneity data stratified by different dimensions: When stratified by age, the 60–65 years subgroup included three studies (*K* = 3), with heterogeneity of I^2^ = 85.3% and τ^2^ = 3152.0139, an effect size of MD = 61.46 (95% CI [−10.24, 133.16]), an evidence quality of GRADE = Low, and *P* = 0.0011; the 65–70 years subgroup included seven studies (*K* = 7), with heterogeneity of I^2^ = 49.4% and τ^2^ = 711.0386, an effect size of MD = 49.86 (95% CI [21.41, 78.31]), an evidence quality of GRADE = Low, and *P* = 0.0654. When stratified by race, the Brazil subgroup included five studies (*K* = 5), with heterogeneity of I^2^ = 74.8% and τ^2^ = 3008.4477, an effect size of MD = 56.82 (95% CI [0.15, 113.49]), an evidence quality of GRADE = Low, and *P* = 0.0032; the China subgroup included five studies (*K* = 5), with heterogeneity of I^2^ = 58.2% and τ^2^ = 596.5158, an effect size of MD = 55.63 (95% CI [26.71, 84.56]), an evidence quality of GRADE = Moderate, and *P* = 0.0485. When stratified by weekly exercise frequency, the subgroup with 3 times per week included seven studies (*K* = 7), with heterogeneity of I^2^ = 71.9% and τ^2^ = 1571.5021, an effect size of MD = 62.84 (95% CI [26.76, 98.91]), an evidence quality of GRADE = Moderate, and *P* = 0.0016; the subgroup with 2 times per week included three studies (*K* = 3), with heterogeneity of I^2^ = 43.8% and τ^2^ = 558.6932, an effect size of MD = 34.42 (95% CI [−6.15, 74.99]), an evidence quality of GRADE = Moderate, and *P* = 0.1685. When stratified by intervention duration, the subgroup with an intervention duration of 8 weeks included three studies (*K* = 3), with heterogeneity of I^2^ = 0% and τ^2^ = 0, an effect size of MD = 108.62 (95% CI [65.16, 152.08]), an evidence quality of GRADE = Very Low, and *P* = 0.9435; the subgroup with an intervention duration of 12 weeks included four studies (*K* = 4), with heterogeneity of I^2^ = 47% and τ^2^ = 594.9868, an effect size of MD = 23.33 (95% CI [−11.57, 58.23]), an evidence quality of GRADE = Low, and *P* = 0.1291; the subgroup with an intervention duration of 24 weeks included three studies (*K* = 3), with heterogeneity of I^2^ = 75.9% and τ^2^ = 1140.8878, an effect size of MD = 56.00 (95% CI [11.52, 100.49]), an evidence quality of GRADE = Low, and *P* = 0.0158. When stratified by exercise duration per session, the subgroup with 90 min per session included only 1 study (*K* = 1), with no relevant heterogeneity data (heterogeneity analysis is not required for a single study), an effect size of MD = 108.10 (95% CI [12.09, 204.11]), an evidence quality of GRADE = Very Low, and no available *P*-value; the subgroup with 60 min per session included six studies (*K* = 6), with heterogeneity of I^2^ = 67.1% and τ^2^ = 1703.5627, an effect size of MD = 47.78 (95% CI [7.42, 88.15]), an evidence quality of GRADE = Moderate, and *P* = 0.0096; the subgroup with 30 min per session included three studies (*K* = 3), with heterogeneity of I^2^ = 75.9% and τ^2^ = 1140.8878, an effect size of MD = 56.00 (95% CI [11.52, 100.49]), an evidence quality of GRADE = Very Low, and *P* = 0.0158.

## Discussion

The network Meta-analysis results showed that Water-Based Exercise had the most significant effect on improving the 6MWD in stable chronic obstructive pulmonary disease (COPD) patients (SUCRA = 98.9, MD = 83.91, 95% CI: 55.98–111.83). Water-based exercise refers to physical activity conducted in an aquatic environment, encompassing various forms of water aerobics. As a complementary exercise modality in rehabilitation programs, this approach offers distinct advantages in minimizing joint stress and enhancing cardiovascular health (90).

Water-based exercise provides unique physiological benefits for patients with COPD. The hydrostatic pressure generated during immersion elevates the diaphragm and facilitates expiration, thereby decreasing dead space in the lungs and helping to reduce dynamic hyperinflation –a major pathophysiological problem in COPD (91). Exercise training in general has also been shown to decrease ventilatory requirements and exercise-induced hyperinflation at submaximal intensities in patients with COPD, supporting the idea that water-based programs can improve ventilatory efficiency (92). In addition, buoyancy reduces weight-bearing and perceived exertion, which lowers the workload on respiratory muscles. Water-based training has been demonstrated to improve both respiratory and peripheral muscle strength (93). The thermoregulatory effect of warm water further enhances cardiovascular responses and oxygen transport during exercise (94). Consistent with these mechanisms, a Cochrane review found that water-based exercise training significantly improved the 6-min walk distance, with a mean difference of 62 m (95). Existing studies have substantiated the efficacy of water-based exercise in rehabilitation, with its unique mechanisms of action potentially elucidating its superior effects on improving the 6MWD (2). Water-based exercise is believed to confer a range of physiological and functional benefits by stimulating peripheral muscle groups and enhancing the efficiency of both cardiovascular and pulmonary systems, thereby improving overall endurance and exercise performance (96, 97). A preliminary study involved 14 stable COPD participants, who were randomly assigned to either a water-based exercise group or a land-based exercise group. Both groups completed an 8-weeks training program, consisting of two sessions per week. Assessments of lung function, respiratory muscle strength, peripheral muscle strength, balance, the 6-min walk test (6MWT), incremental shuttle walk test (ISWT), and endurance shuttle walk test (ESWT) were conducted at baseline and following the program. The results demonstrated that the water-based exercise program significantly enhanced endurance levels, as evidenced by improvements in the endurance shuttle walk test (ESWT) and maximum inspiratory pressure (MIP), indicating enhanced respiratory muscle strength and overall exercise capacity, indirectly influencing the 6MWD (93). The buoyancy of water mitigates the impact of comorbidities on exercise performance and diminishes subjective feelings of fatigue, enabling patients to engage in higher-intensity training. Concurrently, the hydrostatic pressure of water enhances muscle blood flow, thereby improving oxygen delivery and exercise capacity, which significantly boosts endurance and overall exercise performance (98). Moreover, Ian M. Wilcock’s study demonstrated that moderate water temperature positively influences blood circulation and muscle relaxation, facilitating post-exercise recovery and reducing exercise-induced fatigue (99). Consequently, water-based exercise proves beneficial for patients with COPD, whose 6MWD is compromised due to airflow limitation, respiratory muscle fatigue, and decreased exercise tolerance by aiding exhalation through hydrostatic pressure, alleviating dynamic hyperinflation, and decreasing respiratory load. Furthermore, buoyancy reduces joint stress, allowing patients to endure higher-intensity training while enhancing lower limb strength. Additionally, the combined effects of water’s resistance and thermal properties contribute to improved cardiovascular and pulmonary adaptation.

Water-based exercise reduces joint loading through buoyancy, while the resistance of water slows movements to prevent accidents. The combined effects of warm water and hydrostatic pressure also relieve pain and muscle tension, thereby enhancing both safety and comfort (100). These advantages support better adherence, as patients are more likely to continue with low-risk, comfortable exercise (101). In chronic low back pain, aquatic exercise achieved higher recommendation rates than physiotherapy, indicating stronger patient preference (102). For older adults or those with balance issues, water reduces the fear of falling, further increasing their willingness to participate (103).

The results of nonlinear regression and dose-response meta-analysis indicate a clear dose-response relationship between the exercise intervention parameters and the improvement in 6-min walk distance in COPD patients (104). The intervention frequency should be at least three times per week to produce a positive effect, with five sessions per week being the optimal frequency predicted by the model. The duration of each session should be at least 60 min, with 90 min being the optimal point of effect on the trend graph. Guidelines indicate that performing water-based exercise at least three times a week aligns with established recommendations and enhances treatment effects for patients with respiratory diseases (105). This frequency likely activates the body’s adaptive responses, improving cardiovascular function and muscle endurance, as evidenced by enhancements in the 6MWD (106). Furthermore, systematic reviews have summarized the evidence level and recommendation grade for therapeutic aquatic exercise interventions in patients with COPD. These reviews suggest that the optimal duration for a single water therapy session is 90 min. However, other studies have demonstrated that sessions lasting between 30 and 50 min, conducted multiple times per week, can also yield positive outcomes (107, 108). This is attributed to the fact that 60 min of exercise is sufficient to activate and sustain physiological adaptations, thereby improving cardiovascular health and enhancing muscle endurance (109). Regarding intervention duration, 8 weeks is the most effective period. Research indicates that water-based exercise training over 8 weeks significantly improves exercise capacity and quality of life in patients with COPD, including those with physical comorbidities. This training contributes to increased walking distance and reduced symptoms of breathlessness and fatigue (98). An 8-weeks duration provides an adequate timeframe for the body to undergo significant physiological adaptations (110).

The meta-analysis results also showed that a total weekly exercise time of approximately 270 min was the optimal duration predicted by the model, at which the improvement in 6MWD was the greatest. Exercise durations of 90 min per week or less may not lead to significant improvements. Shorter durations of water therapy, specifically less than 90 min per week, may not provide a sufficient stimulus to enhance lung function or exercise capacity significantly. In contrast, the recommendation of a cumulative weekly exercise duration of about 270 min is well-supported by evidence, demonstrating substantial improvements in health status. Moreover, the subgroup analyses showed that: (1) studies with three sessions per week tended to yield greater improvements than those with two sessions, although the subgroup difference did not reach statistical significance (*p* = 0.3049); (2) intervention duration significantly modified the effect (*p* = 0.0111), with the most significant benefit observed in programs lasting 8 weeks; and (3) all session lengths (30, 60, and 90 min) were beneficial, with the most consistent gains seen at 60–90 min per session, but no significant subgroup difference was detected (*p* = 0.5249). These findings align with the nonlinear regression analysis, which suggested an “optimal dose” of approximately three sessions per week, lasting 60–90 min per session, for 8 weeks, totaling around 270 min per week. Together, the consistency between subgroup and nonlinear analyses provides practical reference values for clinical prescription. This underscores that regular and sustained physical activity is crucial for attaining significant health benefits in managing respiratory diseases (111, 112).

Age factor analysis further indicated that older adults aged 61.67 years or younger were more responsive to exercise interventions, showing more substantial improvements. In contrast, older adults, particularly those over 60, may experience slower responses to exercise interventions due to age-related physiological decline, which can adversely affect aerobic capacity, muscle strength, and overall cardiovascular health (113). Although the traditional subgroup analysis did not reveal statistically significant differences between age groups (*p* = 0.7681), the trend was consistent with the nonlinear model, indicating that the relationship between age and intervention efficacy is likely continuous and nonlinear rather than categorical. Taken together, these findings highlight the potential moderating role of age on exercise efficacy in COPD, underscoring the need for future trials to report severity profiles more consistently and to explore age-related differences in training responsiveness further.

The age cutoff of 61.67 years in our meta-regression emerged from the data. While there is no universally accepted clinical threshold at exactly 61.67 years, existing studies support that age starts to moderate physiological and functional adaptation in COPD from approximately 60 to 65 years. At identical isowork rates, aging influences cardiorespiratory adaptations in COPD outpatients (mean age ∼65 ± 7 years), who found that older COPD patients had reduced improvements in ventilatory parameters under submaximal workloads compared to younger ones (114).

The results of the subgroup analysis of the optimal intervention method (WBE) showed no significant differences among various regions (*p* = 0.9708), suggesting that the beneficial effect of Water-Based Exercise is generally consistent across populations from different countries. However, it should be noted that using study location as a proxy for race or ethnicity has limitations, since cultural, healthcare, and environmental factors may differ substantially. Therefore, while our findings support the potential generalizability of WBE across diverse regions, further research with explicit reporting of participant ethnicity and cultural background is warranted.

Chinese traditional mind-body exercises, such as Tai Chi and Qigong, are generally safe due to their low intensity and minimal joint stress, making them suitable for older adults and patients with chronic conditions (115). Evidence shows good adherence, as participants in Tai Chi programs often demonstrate high attendance and a willingness to continue practicing (116). Moreover, patients frequently prefer these practices because they integrate physical movement with breathing, meditation, and relaxation, thereby promoting overall well-being and psychological comfort (117). Chinese traditional mind-body exercises have significantly improved patients’ exercise capacity with COPD (118). Among the studies included, Chinese traditional mind-body training, such as Tai Chi, Baduanjin, and Qigong, served as the primary intervention. At the same time, the control groups received conventional treatment, general exercise, or water-based training. In most studies, the intervention group demonstrated a higher mean 6-min walk distance (6MWD) than the control group, with three studies reporting significant effects. The pre- and post-intervention differences were 64.91, 75.34, and 66.78 m, (46, 48, 56). Traditional mind-body exercises, such as Tai Chi and Qigong, may enhance the 6MWD in COPD patients by improving both physical and cognitive functions, promoting better lung function, and alleviating chronic pain. These practices advocate for a holistic health approach, which may potentially enhance quality of life and reduce the incidence of acute exacerbations (119). The exercises emphasize breath control, relaxation, and gentle movements, which contribute to improved lung mechanics and oxygenation. They can enhance lung function, increase exercise capacity, and promote overall health (118). Consequently, patients with COPD often experience reduced exercise endurance due to respiratory muscle dysfunction, abnormal energy metabolism, and psychological stress. Traditional Chinese mind-body exercises facilitate diaphragmatic activity and enhance ventilation efficiency through coordinated breathing and movement. Furthermore, the gentle nature of these exercises renders them particularly suitable for older patients with COPD, providing better tolerance and potentially more significant benefits than more intensive exercise forms.

## Conclusion

The results of this study suggested that Water-Based Exercise was the optimal exercise intervention for improving 6-min walk distance (6MWD) in COPD patients. Through a nonlinear dose-effect analysis, it was determined that scientifically controlling exercise frequency (at least three times per week, with a predicted optimal value of five sessions), session duration (at least 60 min per session, with a predicted optimal value of 90 min), and intervention duration (8 weeks), while maintaining a total exercise time of about 270 min per week, could yield the best intervention outcomes. Additionally, the study indicated that older adult groups (≤61.67 years) are more likely to benefit from the intervention. This research provides robust data support for developing personalized, evidence-based exercise rehabilitation strategies for patients with COPD.

### Limitations

Although this study provided valuable insights into exercise interventions for COPD patients through meta-analysis, however, several limitations must be acknowledged. Firstly, the inclusion criteria were restricted to randomized controlled trials, which may have excluded pertinent information from other study designs, thereby constraining the generalizability of the findings. Secondly, although the 6MWD serves as a targeted and precise measure of exercise capacity, the analysis could have benefited from including additional outcome indicators, which were not considered in this study. Furthermore, given that the sole outcome measure utilized was the 6MWD, expressed in meters, and that some studies had limited sample sizes, the effect sizes and confidence intervals reported in this study were notably large, potentially leading to instability in the effect estimates. However, information on COPD severity (e.g., GOLD stage) and dyspnea levels (e.g., MMRC scores) was not consistently reported across studies, which may limit the generalizability of our findings to patients at different stages of the disease. Several included trials had small sample sizes (<20 participants per group), which may reduce the stability and generalizability of the pooled effect estimates. Future research should aim to increase sample sizes, incorporate more high-quality studies, and investigate the long-term effects of various exercise interventions to validate and expand upon the findings of this study.

## Data Availability

The raw data supporting the conclusions of this article will be made available by the authors, without undue reservation.
