# Supplementary material for: Effects of various exercise therapies on 6-min walk distance in patients with stable chronic obstructive pulmonary disease: a systematic review and network meta-analysis
Source: Front Med (Lausanne). 2025 Oct 29;12:1668578. doi: 10.3389/fmed.2025.1668578 (PMC12605192; doi:10.3389/fmed.2025.1668578)

1.Supplementary Table S1. Search strategies

Database	Search strategy
PubMed	("Pulmonary Disease, Chronic Obstructive"[Mesh] OR COPD OR "Chronic Obstructive Pulmonary Disease" OR "Chronic Obstructive Lung Disease" OR "Chronic Obstructive Airway Disease" OR "Stable COPD" OR "Stable Chronic Obstructive Pulmonary Disease") AND ("Exercise Therapy"[Mesh] OR Exercise OR "Physical Activity" OR Training OR "Aerobic Training" OR "Resistance Training" OR "Pulmonary Rehabilitation" OR Rehabilitation OR Sports) AND ("Walking Test"[Mesh] OR "6-minute walk test" OR "6-minute walk distance" OR "6MWT" OR "Six Minute Walk" OR "Endurance Walk Test" OR "Walking Capacity") AND ("Randomized Controlled Trial"[Publication Type] OR "Controlled Clinical Trial"[Publication Type] OR RCT OR "Randomized Trial")
PsycINFO	(DE "Chronic Obstructive Pulmonary Disease" OR COPD OR "Chronic Obstructive Pulmonary Disease" OR "Chronic Obstructive Lung Disease" OR "Stable COPD") AND (DE "Exercise" OR "Exercise Therapy" OR "Physical Activity" OR Rehabilitation OR "Pulmonary Rehabilitation" OR "Aerobic Training" OR "Resistance Training" OR Training) AND ("6-minute walk test" OR "6-minute walk distance" OR "6MWT" OR "Six Minute Walk" OR "Walking Capacity" OR "Endurance Walk Test") AND (DE "Randomized Controlled Trials" OR "Controlled Clinical Trial" OR RCT OR "Randomized Trial")
Web of Science	TS = (COPD OR "Chronic Obstructive Pulmonary Disease" OR "Chronic Obstructive Lung Disease" OR "Chronic Obstructive Airway Disease" OR "Stable COPD" OR "Stable Chronic Obstructive Pulmonary Disease") AND TS = ("Exercise Therapy" OR Exercise OR "Physical Activity" OR Training OR "Aerobic Training" OR "Resistance Training" OR "Pulmonary Rehabilitation" OR Rehabilitation OR Sports) AND TS = ("6-minute walk test" OR "6-minute walk distance" OR 6MWT OR "Six Minute Walk" OR "Walking Capacity" OR "Endurance Walk Test") AND TS = ("Randomized Controlled Trial" OR "Controlled Clinical Trial" OR RCT OR "Randomized Trial")
Cochrane Library	([mh "Pulmonary Disease, Chronic Obstructive"] OR COPD OR "Chronic Obstructive Pulmonary Disease"

	OR "Chronic Obstructive Lung Disease" OR "Chronic Obstructive Airway Disease"<br>OR "Stable COPD" OR "Stable Chronic Obstructive Pulmonary Disease")<br>AND ([mh "Exercise Therapy"] OR Exercise OR "Physical Activity" OR Training OR "Aerobic Training"<br>OR "Resistance Training" OR "Pulmonary Rehabilitation" OR Rehabilitation OR Sports)<br>AND ([mh "Walking Test"] OR "6-minute walk test" OR "6-minute walk distance" OR 6MWT<br>OR "Six Minute Walk" OR "Endurance Walk Test" OR "Walking Capacity")<br>AND ([mh "Randomized Controlled Trial"] OR "Controlled Clinical Trial" OR RCT OR "Randomized Trial")
CNKI	主题 = ("慢性阻塞性肺疾病" OR "慢阻肺" OR COPD OR "慢性阻塞性肺气肿" OR "稳定期 COPD" OR "稳定期慢性阻塞性肺疾病")<br>AND 主题 = ("运动" OR "运动疗法" OR "运动训练" OR "康复训练" OR "运动康复" OR "体力活动" OR "锻炼" OR "有氧训练" OR "抗阻训练" OR "肺康复")<br>AND 主题 = ("6 分钟步行试验" OR "6 分钟步行距离" OR "六分钟步行试验" OR "步行能力" OR "耐力步行试验" OR 6MWT)<br>AND 主题 = ("随机对照试验" OR "随机临床试验" OR RCT OR "对照研究")

2.Supplementary Table S2.Subgroup Analysis Results of WBE

Dimensionality	sort	K	N	I^2	Effect model	MD and 95%CI	GRADE	P
Race								
	Brazil	5	132	74.8%	Random	56.82(0.15,113.49)	Low	0.0032
	China	5	184	58.2%	Random	55.63(26.71,84.56)	Moderate	0.0485

Age								
	60−65	3	105	85.3%	Random	61.46(-10.24,133.16)	Low	0.0011
	65−70	7	211	49.4%	Random	49.86(21.41,78.13)	Low	0.0654
Motion frequency/week								
	3	7	220	71.9%	Random	-0.38(-0.73, -0.02)	Moderate	0.036
	2	3	96	43.8%	Random	-2.33(-3.52, -1.15)	Moderate	0.001
Intervention Time								
	8	3	65	0%	Random	108.62(65.16,152.08)	Very Low	0.9435
	12	4	127	47%	Random	23.33(-11.57,58.23)	Low	0.1291
	24	3	124	75.9%	Random	56.00(11.52,100.49)	Low	0.0158
Exercise Time								
	90	1	24	*	Random	108.10(12.09,204.11)	Very Low	*
	60	6	168	67.1%	Random	47.78(7.42,88.15)	Moderate	0.0096
	30	3	124	75.9%	Random	56.00(11.52,100.49)	Very Low	0.0158

3.Supplementary Figure S1.Forest Plot of WBE

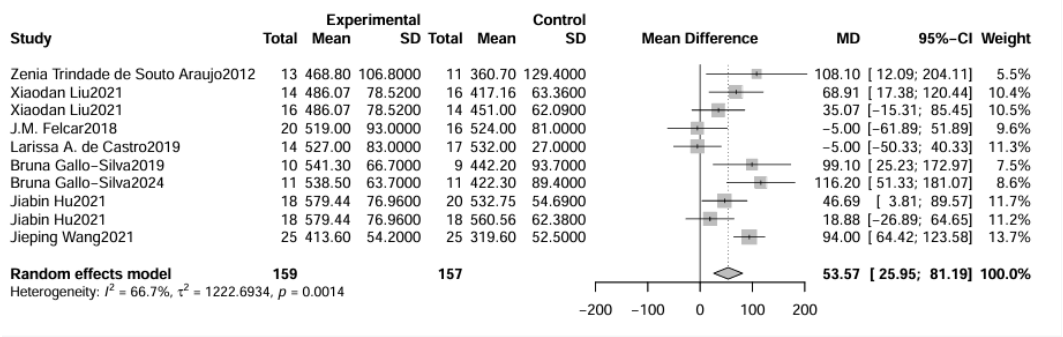

4.Supplementary Figure S2.Results of Age Subgroup Analysis for WBE

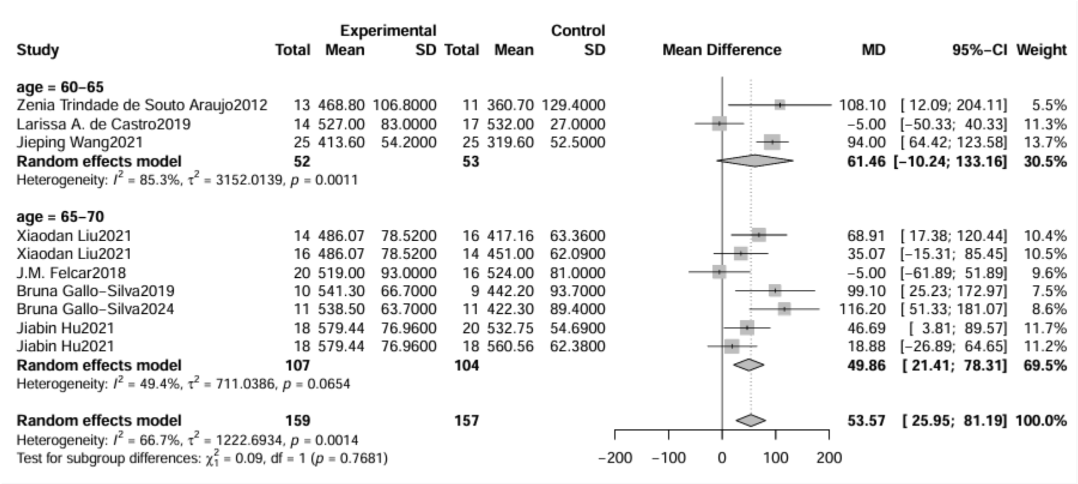

5..Supplementary Figure S3.Results of Race Subgroup Analysis for WBE

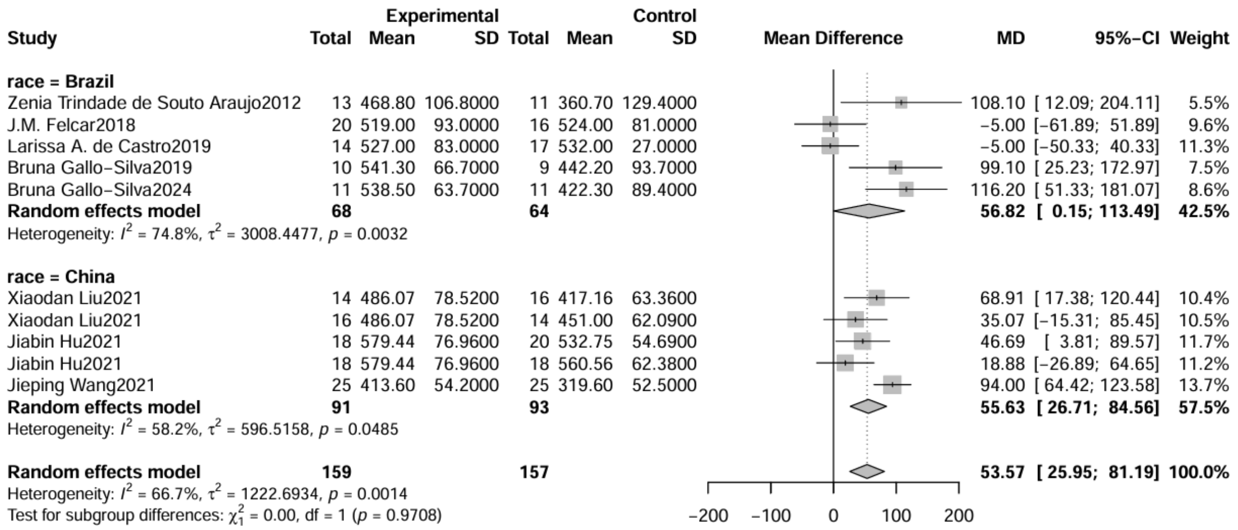

6.Supplementary Figure S4.Results of Exercise Time Subgroup Analysis for WBE

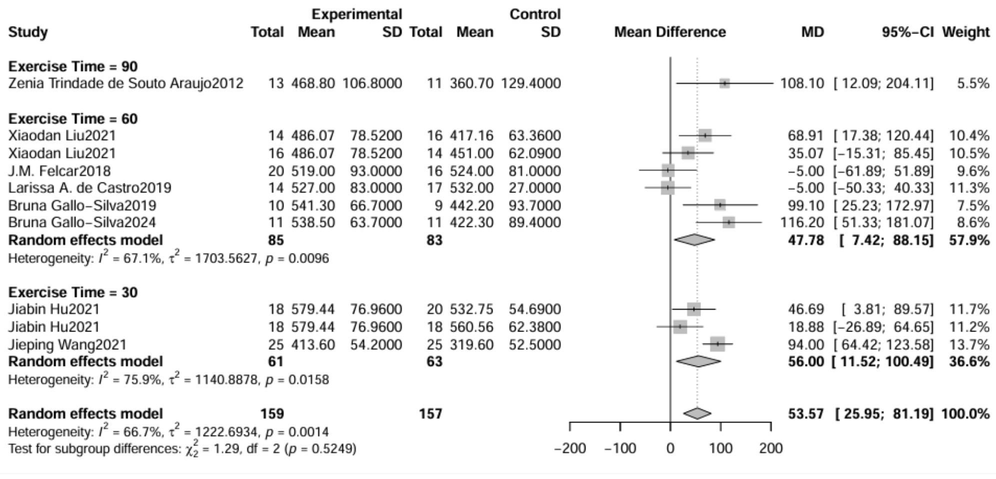

7.Supplementary Figure S5.Results of Intervention Time Subgroup Analysis for WBE

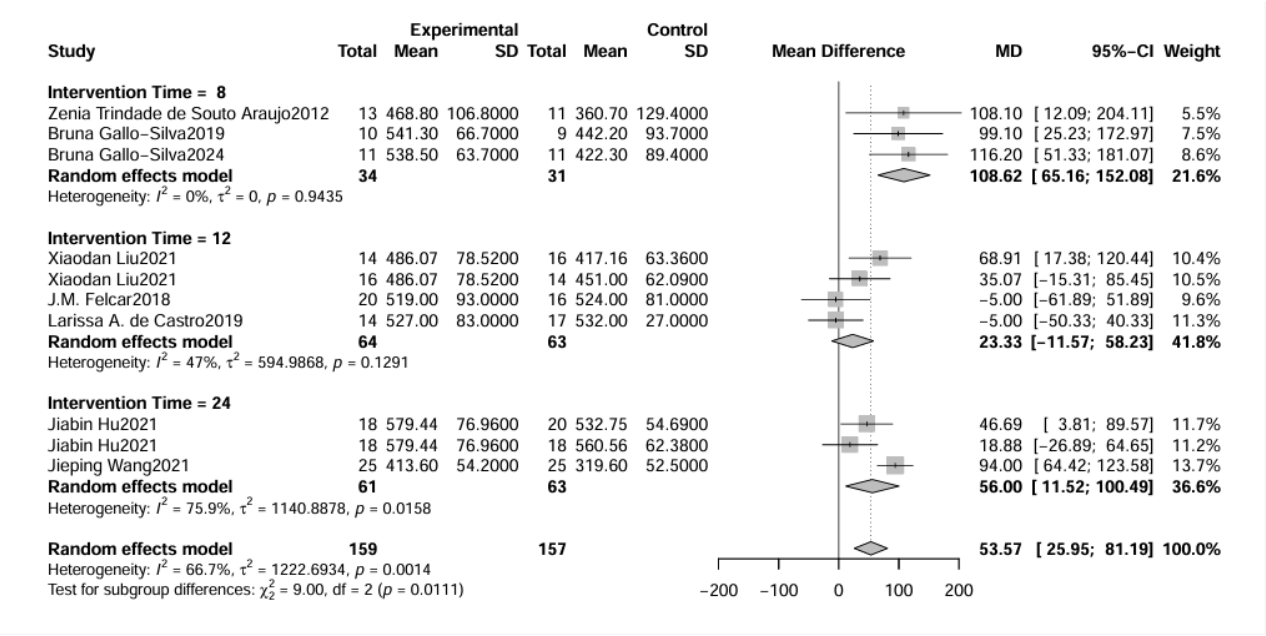

8.Supplementary Figure S6.Results of Motion Frequency Subgroup Analysis for WBE

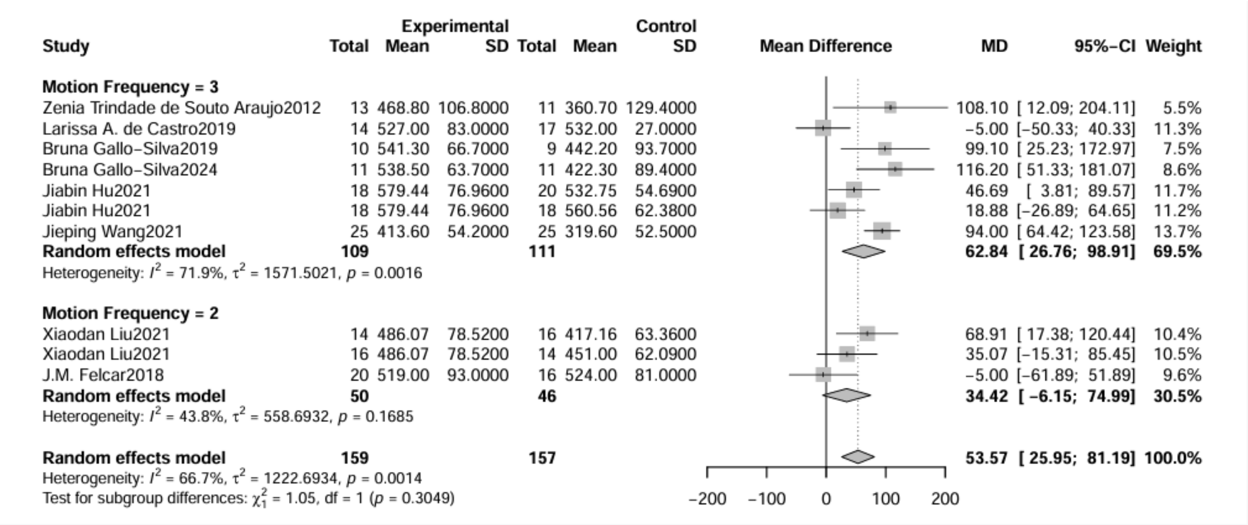

Supplement: Supplementary file 1 [file Data_Sheet_1.pdf]
